# A Treatise on Materia Medica

**Published:** 1890-02

**Authors:** 


A TREATISE ON MATERIA MEDICA, PHARMACOLOGY, AND THERAPEUTICS. By John V. Shoemaker, A. M., M. D., and John Aulde, M. D. In two volumes, Vol. I. Philadelphia and London: F. A. Davis, Publisher.

Although this work is intended for our allopathic brethren, a glance at its contents will show that we may profit by much that it contains. The authors of the work deserve high praise for the ability and labor shown in its pages. They have here brought together much that is otherwise found only in a number of volumes, and they thus place the medical profession under obligations. The authors are professors in the Medico-Chirurgical College, of Philadelphia, and, of course, see things only through allopathic spectacles, but they are more progressive than the average allopath, and if they keep on they will find themselves viewing therapeutics in a new light.

